# SURGICAL PROCEDURES PROLONG AMBULATION IN PATIENTS WITH DUCHENNE MUSCULAR DYSTROPHY

**DOI:** 10.1590/1413-785220253302e287732

**Published:** 2025-10-13

**Authors:** DAVID GONÇALVES NORDON, FELIPE CRUZ CAETANO DOS REIS, CARLOS ALBERTO DOS SANTOS, ADILSON DE PAULA, MARIA BERNADETE DUTRA DE RESENDE, PATRICIA MORENO GRANGEIRO

**Affiliations:** 1. Universidade de Sao Paulo, Faculdade de Medicina, Hospital das Clinicas (HCFMUSP), Instituto de Ortopedia e Traumatologia, Centro Integrado de Neuro-ortopedia, Sao Paulo, SP, Brazil.

**Keywords:** Duchenne Muscular Dystrophy, Treatment, Tenotomies, Distrofia Muscular de Duchenne, Tratamento, Tenotomia

## Abstract

**Objective:**

The aim of this study was to evaluate the effect of surgical procedures on maintaining ambulation for Duchenne patients.

**Methods:**

This retrospective cohort study evaluated 35 patients for whom surgery was recommended at our institution from 2012 to 2020.

**Results:**

Twenty-seven patients were operated on before gait loss, and eight after. In this study, surgical treatment allowed recovery and prolongation of gait for 38.6 months, on average. The sooner the surgery was performed, the better the results were; logistic regression analysis showed that each day of delay after gait loss decreased the chances of success by 0.2%. The optimal interval for intervention was up to 12 months after gait loss.

**Conclusion:**

Our results thus corroborate the evidence that surgical interventions are beneficial for these patients and suggest a not previously described time window for achieving better outcomes. Level of Evidence lll; Retrospective, Comparative Study of Surgical Interventions.

## RESUMO

**Objetivo:**

O objetivo deste estudo foi avaliar o efeito de procedimentos cirúrgicos para manutenção da marcha em pacientes com Duchenne.

**Métodos:**

esta coorte retrospectiva avaliou 35 pacientes para os quais cirurgia foi recomendada na nossa instituição entre 2012 e 2020.

**Resultados:**

vinte e sete pacientes foram operados antes da perda de marcha, e oito pacientes, depois. Neste estudo, o tratamento cirúrgico permitiu recuperação ou manutenção da marcha por 38,6 meses, em média. Quanto mais cedo foi realizada a cirurgia, melhores os resultados. Análise de regressão logística mostrou que cada dia de atraso após perda da marcha diminuiu as chances de sucesso por 0,2%. O intervalo ótimo para intervenção foi de 12 meses após o paciente perder a marcha.

**Conclusão:**

nossos resultados assim corroboram para a evidência de que intervenções cirúrgicas são benéficas para estes pacientes e apresentam uma janela de tempo até então não definida para melhores resultados. Nível de Evidência lll; Estudo Retrospectivo, Comparativo de Intervenções Cirúrgicas.

## INTRODUCTION

Duchenne muscular dystrophy (DMD) is a genetic disease related to the X chromosome, that affects approximately 1 in every 20,000 boys.^1,2^ The genetic mutation for DMD compromises the production of dystrophin, what leads to progressive muscular weakness, loss of developmental motor milestones, and loss of gait at around 13 years of age.^3^

The current main pharmacological treatment consists of glucocorticoids (GC), to be prescribed as soon as patients reach their motor developmental plateau, at around 5 years of age.^4,5^ Physical therapy is directed towards the maintenance of strength and prevention of muscular and tendinous contractures, which are common as the disease progresses. Gait loss is directly related to disease progression, as wheelchair-bound patients present a faster decline in the course of the disease.^3^ Orthopedic surgical interventions intended to release contractures may be used for preventative, rehabilitative, or palliative purpose.^6^ The hip flexors and abductors, knee flexors, and the Achilles tendon may be addressed during treatment, all of which, when contracted, promote characteristic deformities, and impair gait.

There are some controversies regarding the effectiveness of surgical procedures for the treatment of contractures and gait maintenance: while several studies present good results for multilevel surgeries,^7-13^ a specialists’ consensus does not recommend multilevel procedures, restricting indications to Achilles tendon lengthening.^14^

To date, there is a lack of studies that have evaluated the effectiveness and prognostic impact of surgical treatment for patients with DMD in Brazil. Therefore, the objective of this study was to evaluate the direct effects of surgical procedures (maintenance, recovery, or deterioration) on the gait of patients with DMD that were operated on at our institution.

## MATERIAL AND METHODS

Charts of patients with DMD followed by our neuro-orthopedics outpatients’ clinics were retrospectively reviewed. This study was approved by the Hospital’s Ethics Committee (CAAE 29523920.0.0000.0068).

### Population

The study was conducted at the Orthopedics Department of a tertiary hospital. All patients submitted to surgery or on a waiting list for surgery during the period from January, 2012 to December, 2020 were analyzed for inclusion, thus defining a convenience sample.

Inclusion criteria were: confirmed diagnosis of DMD (either by genome or muscle biopsy); minimum 24 months of post-operative follow-up; complete chart, containing data concerning the time of gait interruption, date of surgery, description of the performed surgical procedure, retainment or not of gait ability after surgery, and occurrence of postoperative complication. In cases where the chart was incomplete, the patient’s family or caregiver was interviewed.

Exclusion criteria consisted of: muscular dystrophies other than Duchenne’s; incomplete chart or unreliable information; post-operative follow-up shorter than 24 months. Age was not a criterion for inclusion or exclusion.

Included patients were divided into two groups: patients in group 1 had stopped walking before surgery, and patients in group 2 were still walking before surgery. If present, information on medication in use was included.

At our institution, surgery is recommended for all DMD patients who present tendon contractures, regardless of the patient’s ambulatory status. Surgery might be indicated even for wheelchair bound patients, for comfort and/or hygienical purposes, according to parents’ or legal guardians’ desire.

The Covid-19 pandemic had an important impact in our services, and elective surgical procedures were interrupted for over 15 months, from March, 2020 on.

This generated an unexpected group: patients with tendon contractures who could not be operated on. For these patients, we collected data on their ambulatory status.

### Statistical Analysis

In this study, a good outcome after surgery was considered when the patient retained or recovered the ability to walk. Three clinical questions were addressed:

1. Is it necessary to intervene before losing gait?
2. Is there a relationship between age and a good surgical outcome?
3. Is it more effective to perform the orthopedic procedure less than 12 months before the patient stopped walking?

Based on our limited sample size, we approached the first question using the Monte Carlo Sampling Algorithm,^15^ then we applied a Fisher Exact Test.^16^

To answer the second question, we applied a T-Test^17^ and performed a logistic regression analysis.^18^

For the third question, we applied a Hypothesis Test, a T-Test, and a Logistic Regression.

## RESULTS

**Figure 1:**
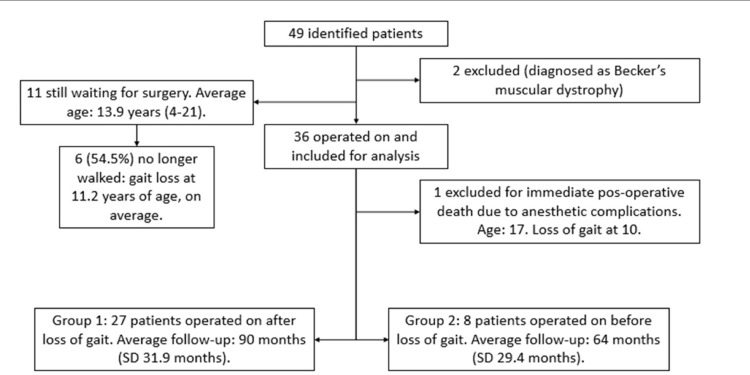
Distribution of the 49 identified patients.

A total of 49 patients were identified. Their outcomes and distribution are presented in Figure 1 and Tables 1 and 2.

**Table 1:** Information on operated DMD patients (G1).

Patient number	Age when ceased walking (in years)	Interval between gait loss and procedure (in months)	Age at surgery (in years)	Single (S) or Multi (M) level surgery	Gait recovery after procedure (Yes/No)	Interval until new disability (in months)	Glucocorticoids use (Yes/No/NA = Not available)
1	10	120	19	M	No	NA	Yes
2	10	11	11	M	No	NA	Yes
3	8	36	10	M	No	NA	NA
4	7	36	10	M	No	NA	NA
5	9	120	9	M	No	NA	Yes
6	12	5	12	S	No	NA	Yes
7	11	12	12	M	No	NA	Yes
8	12	12	13	M	No	NA	NA
9	11	24	13	M	No	NA	NA
10	10	7	11	M	No	NA	Yes
11	17	24	19	M	No	NA	NA
12	11	9	12	M	No	NA	NA
13	10	24	11	M	No	NA	Yes
14	8	12	9	M	Yes	3	Yes
15	10	12	11	M	No	NA	NA
16	13	7	13	S	No	NA	NA
17	11	7	11	M	No	NA	NA
18	12	108	21	M	No	NA	Yes
19	10	17	11	M	No	NA	Yes
20	12	60	17	M	No	NA	Yes
21	10	24	12	M	Yes	120	Yes
22	13	12	14	M	Yes	30	Yes
23	8	2	8	M	Yes	30	NA
24	10	8	11	M	Yes	42	Yes
25	7	6	8	M	Yes	7	NA
26	8	9	9	M	Yes	Retains gait	NA
27	9	7	9	S	Yes	Retains gait	Yes

**Table 2:** DMD patients operated on before gait loss (G2).

Patient number	Age at surgery (in years)	Single (S) or Multi (M) level surgery	Gait maintained after procedure? (Yes/No)	Interval until new disability (in months)	Glucocorticoids use (Yes/No/NA = Not available)
1	7	M	Yes	Retains gait	Yes
2	11	S	Yes	Retains gait	NA
3	12	S	Yes	Retains gait	Yes
4	23	M	Yes	Retains gait	Yes
5	10	M	No	NA	NA
6	12	M	Yes	11	Yes
7	9	M	Yes	3,5	Yes
8	14	S	No	NA	Yes

Single-level surgeries consisted of either uni- or bilateral Achilles’ tendon lengthening. Multi-level surgeries consisted of unilateral or bilateral tenotomies at the hips (iliopsoas, fascia lata, gluteus), knees (ischiotibial muscles, femoral biceps), and ankles (Achilles’ tendon), and were performed as required for adequate contracture release.

### Timing of surgery in relation to gait loss and its effects

Groups 1 and 2 were compared for this analysis. There was a statistically significant difference concerning surgical results (gait maintenance/recovery) between both groups, in favor of the group that was still walking at the moment of surgery (29.6% recovered gait in Group 1, and 75% maintained gait in Group 2, p = 0.04).

### Age at the procedure and gait recovery after the procedure

**Figure 2:**
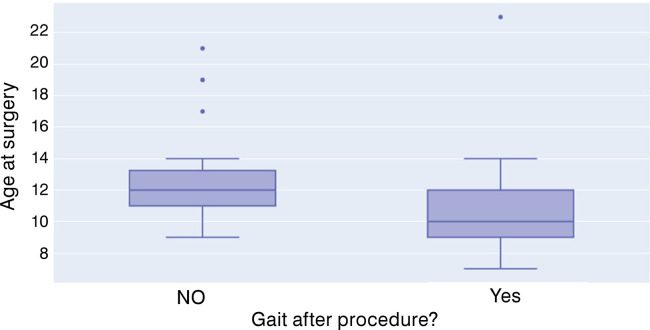
Age distribution of patients in Group 1 at the time of procedure and occurrence of gait recovery.

Within group 1, patients who recovered gait after the procedures were significantly younger than those who did not (average 10 years vs 12.9 years, p = 0.), as shown in Figure 2. Logistic regression suggested that the younger the patient was, the higher his chance of recovering gait, increasing by 5% per year younger (considering the sample age interval of 5 to 23 years old).

### Interval between gait loss and surgery

**Figure 3:**
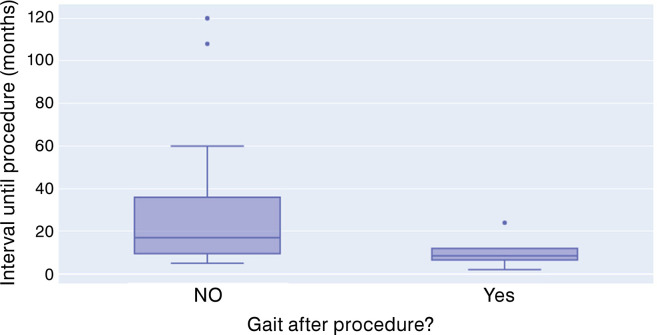
Distribution of the interval between loss of gait and surgery between in group 1 and the outcome (gait recovery).

Patients from group 1 were evaluated concerning the interval, in months, between gait loss and surgical procedure, as well as the surgical outcome (Figure 3). Those who recovered gait after the procedure had a shorter interval than those who did not recover gait (10 vs 34.26 months, p= 0.001).

### Time limit for intervention after the loss of gait

Different intervals were tested (3, 6, and 12 months) as the defining limit for the loss of gait. Procedures performed before 12 months of gait loss presented statistically significant better results than procedures performed after 12 months (p = 0.014). By logistic regression, it was possible to calculate that for each month of delay in surgery, chances of success (gait recovery) decreased by 6% in average; and each day of delay represented a 0.2% decrease in chances to recover gait.

**Figure 4:**
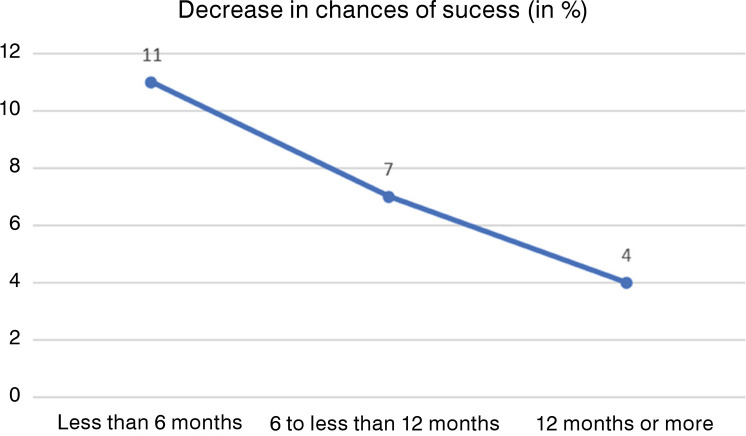
Monthly decrease in chances of success (gait recovery) after surgery, according to time frame after gait lost.

When comparing different intervals (Figure 4), it was possible to observe that chances of success were higher in the first six months and each month of delay represented an 11% decrease in success rate. In the following six months, the decrease of chance of success was less intense (from 6 to less than 12 months, each month of delay represented a 7% decrease), and, finally, it was the smallest in the last interval (4% for each month of delay).

Gait recovery or maintenance depends on the patient’s inherent chance of success, which is directly related to age and gait status. That is, chances of success are even slimmer for the older, non-ambulatory patients who wait longer for surgery.

### Surgical procedure technique: single- or multi-level

Groups 1 and 2 were evaluated both separately and combined. In total, of those patients who underwent multi-level surgery, 10 recovered gait, and 18 did not (64,3%). Of those who underwent single-level surgery, four recovered gait, and two did not (33%) (p=0.34).

### Glucocorticoid use

This information was missing in 14 patients’ charts and remained unknown even after direct questioning, therefore it was impossible to analyze GC’s influence on gait. However, all patients with available information (21 out of 35) used GC, what is in accordance with our institutional protocol.

### Average extended time of gait after surgery

For those patients who recovered gait after surgery, the average extended time of gait was of 38.6 months before a new disability, which occurred at 14.9 years of age, on average. Of the eight patients operated on before losing gait, two ceased walking after surgery, two retained gait for 3.5 and 11 months (average 7.2) before a new disability, and four retained gait until the end of follow-up (30 months on average). Gait maintenance could be even longer in the patients from both groups who were still walking at the end of follow-up.

## DISCUSSION

Surgical recommendations for DMD are uncertain in the available literature. Our study demonstrated that orthopedic procedures may be of benefit for patients with DMD, even after gait loss (up to less than 12 months). This is the first study to suggest a window of opportunity for this type of intervention in DMD.

Several studies have highlighted the importance of orthopedic surgery for DMD, including classical studies from Rideau^19^ and Forst.^9^ Their procedures of choice were the severing of the tendons at the iliac spine (*tensor fasciae lata, sartorius*, and *rectus femoris*), resection of the gluteal fascia, excision of the iliotibial tract, tenotomy of the medial hamstrings, chevron fasciotomy of the lateral knee flexors, lengthening of the Achilles’ tendon, and transfer of the posterior tibialis, if necessary.

These techniques were reproduced in following studies by Forst himself^10^ and Weiss.^8^ These classic procedures are, however, slightly different from the procedures used in our institution. Our choice of selectively treating muscle contractures was used to minimize strength loss. By using intraoperative evaluation, only muscles directly causing contraction were addressed, just enough to recover the range of movement. That is, not every muscle described by Forst was lengthened in our patients. Results were, nevertheless, positive, and the optimal surgical technique is yet to be defined.

Several studies have reported positive results of orthopaedic intervention on prolonging gait in DMD. In a retrospective study of 86 patients by Weiss’^8^ they were divided into four groups: the first group, of 10 patients, was operated on and received no GC. They lost ambulation by the age of 11.1 years on average. A second group of 32 patients who received no treatment (neither surgery, nor GC), stopped walking at the average age of 9.6 years. A third group of 23 patients was treated only with GC, and lost ambulation at the average age of 11.2 years (what is the exact same age of our non-surgical group).

Finally, in this study,^8^ a fourth group of 21 patients was treated with GC and operated on, and they preserved gait until the age of 14.9 years, the same average age that our patients ceased walking after the same treatment. Therefore, our study corroborates with the previous findings from Weiss, in 2020.^8^

Forst’s study^10^ was also positive concerning the importance of surgical procedures: early surgery (that is, before gait cessation) has prolonged gait ability for 15 months on average. There is a remarkable difference between the findings from Forst’s study and ours, for in Forst’s study patients did not use GC, since it was not the gold standard by then. Surgery (combined with GC) has prolonged gait for 38.6 months in our study.

Smith^12^ has presented some positive results for the surgery on patients who did not use GC as well. Of the 29 patients included in the study, 14 were still walking before surgery, and 15 were not. All patients recovered their gait ability after the procedure, and gait was prolonged for another 3 years in the first group, and 2.5 years in the second group, thus presenting findings slightly better than the group from Weiss’ study^8^ that was not using GC. Smith’s technique was different from the other studies, since it performed only percutaneous tenotomies. That might explain the difference in the results since smaller procedures are considered to generate less loss of strength.

Goertzen^20^ also presented positive results after the orthopedic intervention. Procedures were performed at the average age of 6.1 years, what is considerably early in comparison to other studies, and patients were followed up until 9.7 years of age. A longer follow-up would have been more appropriate (since they usually stop walking at around 11 years of age).

In contrast, Griffet’s study^7^ was the first (and only) one to present “negative” results. It consisted of a retrospective analysis of cases operated between 1990 and 2009. Out of the 17 DMD cases operated on at an average age of 12.4 years, three were still walking before surgery and presented a gait prolonged for 8 more months (6-12 months), on average. The other 14 patients were unable to walk at the moment of surgery and presented no recovery afterwards, no matter if the loss was “a little before” or “much before” the surgery (the exact period of time was not described). Surgical technique and indication were very similar to ours. The authors conclude that procedures should be indicated when the patient is about to lose gait, with the intention to recover standing ability, more than recovering the ability to ambulate. Their results, as ours, once again highlight the role of age, when considering surgical treatment for DMD.

Despite so much evidence, the current consensus on the treatment of DMD^14^ affirms that surgical treatment should only be indicated during the walking phase, and exclusively for Achilles’ tendon lengthening, if it compromises gait. This consensus, however, is based on specialists’ opinions and disregards several decades of evidence in favor of surgical treatment in DMD, as shown above.

Our findings, along with previous studies, indicate that patients with DMD may benefit from surgical procedures of multilevel lengthening or tenotomy for the preservation or recovery of gait, with further improved results if the patients are concomitantly using GC.^8^ Our study also presents a time limit for this procedure – ideally, before 12 months of time after gait loss – and, by logistic regression, estimates how much each month of delay compromises surgical results.

### Study limitations

This study was a retrospective cohort; therefore, there is a limitation in the data that can be obtained in group comparison. Even though the lack of data concerning GC does not compromise the results, it decreases the possibilities of analysis between different groups. The sample size was small, which limits the strength of conclusions that can be drawn, particularly in subgroup analyses. Nevertheless, the sample size was considerable when compared to similar studies. It is representative of the population that is treated in our outpatient clinic; however, more studies are necessary (ideally, clinical trials) to help in defining new guidelines to optimize outcomes for this patient cohort.

## CONCLUSION

Surgical treatment for DMD extended gait for 38.6 months, on average in our patient cohort. The sooner it is performed, the better the results. The outcomes for single or multilevel surgeries did not show statistical differences. Surgery may be performed after the loss of gait and still have positive results, however, the time limit for intervention is less than 12 months. Each day of delay after gait loss decreases the chances of success by 0.2%.
